# Draft Genome Sequence of *Methanobacterium* sp. Maddingley, Reconstructed from Metagenomic Sequencing of a Methanogenic Microbial Consortium Enriched from Coal-Seam Gas Formation Water

**DOI:** 10.1128/genomeA.00082-12

**Published:** 2013-01-24

**Authors:** Carly P. Rosewarne, Paul Greenfield, Dongmei Li, Nai Tran-Dinh, David J. Midgley, Philip Hendry

**Affiliations:** a CSIRO Animal, Food and Health Sciences, North Ryde, NSW, Australia; b CSIRO Mathematics, Informatics and Statistics, North Ryde, NSW, Australia

## Abstract

The draft genome of *Methanobacterium* sp. Maddingley was reconstructed from metagenomic sequencing of a methanogenic microbial consortium enriched from coal-seam gas formation water. It is a hydrogenotrophic methanogen predicted to grow using hydrogen and carbon dioxide.

## GENOME ANNOUNCEMENT

A sample of coal-seam gas formation water was collected from Maddingley, Victoria, Australia (37°49′54″S, 144°25′23″E). It was sourced from a borehole intersecting a brown coal seam at approximately 90 m subsurface. The temperature of the formation water was 22°C, with a pH of 6.9, and electrical conductivity (EC) of 3,515 (µs·cm^-1^), and it contained ~0.5% organic matter. An anaerobic microbial enrichment was established in mMSY medium (1) at 25°C and was subsequently shown to use brown coal as a sole carbon source for methanogenesis (data not shown). Metagenomic DNA from the enrichment culture was extracted using the Meta-G-Nome isolation kit (Epicentre Biotechnologies), sequenced using Illumina HiSeq (100-bp PE library) and assembled using Velvet 1.1.07 (*k* = 41). The resulting scaffolds were separated into individual genome bins based on characteristic trinucleotide frequency signatures and equivalent read coverage.

One of the draft genome bins is representative of an archaeon designated *Methanobacterium* sp. Maddingley. It shares 96% sequence identity over the 16S rRNA gene (1,080/1,122 residues) with *Methanobacterium* sp. AL-21, a mesophilic methanogen isolated from freshwater (NCBI genome sequence accession no. CP002551). The draft genome bin contains a 2,420,154-bp draft genome comprising 104 large contigs (>200 bp) with a mean contig size of 23,271 bp, median of 6,587 bp, N50 of 55,867 bp, and a maximum length of 201,691 bp. The mean GC content of the genome was 38.6%. All of the contigs in the genome bin had an approximate depth of coverage of 301× and comprised approximately 11% of the total metagenomic reads. Annotation was performed using IMG ER (Integrated Microbial Genomes Expert Review) (2), which predicted a total of 2,411 protein-coding genes and 45 structural RNAs. The annotated draft genome was scrutinized for the presence of single-copy genes to confirm that the genome bin comprised contigs from a single organism.

The genus *Methanobacterium* consists mostly of mesophilic methanogens from diverse environments following reclassification of many thermophilic isolates into the genus *Methanothermobacter* (3). *Methanobacterium* sp. Maddingley is predicted to grow on H_2_ and CO_2_ as methanogenic substrates. In the coal-degrading anaerobic enrichment culture, we hypothesize that fermentation products released by *Clostridium* sp. Maddingley (4) may support growth of *Methanobacterium* sp. Maddingley. Further investigation of potential syntrophic interactions is important for enhancing microbial coal-seam gas methanogenesis *in situ*.

### Nucleotide sequence accession numbers.

This Whole Genome Shotgun project has been deposited at DDBJ/EMBL/GenBank under the accession no. AMGN00000000. The version described in this paper is the first version, AMGN01000000.
